# Measuring Spatial Ability for Talent Identification, Educational Assessment, and Support: Evidence from Adolescents with High Achievement in Science, Arts, and Sports

**DOI:** 10.11621/pir2021.0205

**Published:** 2021-06-30

**Authors:** Anna V. Budakova, Maxim V. Likhanov, Teemu Toivainen, Alexey V. Zhurbitskiy, Elina O. Sitnikova, Elizaveta M. Bezrukova, Yulia Kovas

**Affiliations:** a International Center for Research in Human Development, Tomsk State University, Tomsk, Russia; b Sirius University of Science and Technology, Sochi, Russia; c Department of Psychology, Goldsmiths, University of London, London, UK

**Keywords:** education, educational streaming, factor analysis, investment of effort, gifted children, reliability, spatial ability

## Abstract

**Background:**

Spatial ability (SA) is a robust predictor of academic and occupational achievement. The present study investigated the psychometric properties of 10 tests for measuring of SA in a sample of talented schoolchildren.

**Objective:**

Our purpose was to identify the most suitable measurements for SA for the purpose of talent identification, educational assessment, and support.

**Design:**

Our sample consisted of 1479 schoolchildren who had demonstrated high achievement in Science, Arts, or Sports. Several criteria were applied to evaluate the measurements, including an absence of floor and ceiling effects, low redundancy, high reliability, and external validity.

**Results:**

Based on these criteria, we included the following four tests in an Online Short Spatial Ability Battery “OSSAB”: Pattern Assembly; Mechanical Reasoning; Paper Folding; and Shape Rotation. Further analysis found differences in spatial ability across the three groups of gifted adolescents. The Science track showed the highest results in all four tests.

**Conclusion:**

Overall, the study suggested that the *Online Short Spatial Ability Battery (OSSAB)* can be used for talent identification, educational assessment, and support. The analysis showed a unifactorial structure of spatial abilities. Future research is needed to evaluate the use of this battery with other specific samples and unselected populations.

## Introduction

Spatial ability can be defined as the ability to generate, retain, retrieve, and transform visual images (Lohman, 1996). It plays an important role in academic performance (Kell, Lubinski, Benbow, & Steiger, 2013; Tosto et al., 2014; Xie et al., 2020), particularly in interest and accomplishment in Science, Technology, Engineering, and Mathematics (STEM) fields (Super & Bachrach, 1957; Wai, Lubinski, & Benbow, 2009; Li & Wang, 2021).

For example, individuals from Project Talent (Flanagan et al., 1962) with more pronounced spatial ability (compared to verbal ability) were more involved in math and science courses in high school (Wai et al., 2009). They were also more likely to choose the STEM fields for future education, while those with the opposite pattern (verbal ability advantage over spatial) were more likely to choose educational programs and careers focused on education, humanities, and social sciences.

Moreover, it appears that the likelihood of obtaining an advanced degree in STEM (from a BSc to a PhD) increases as a function of spatial ability: 45% of all those holding STEM PhDs scored within the top 4% on spatial ability 11 years earlier; and nearly 90% of all those holding STEM PhDs were in top 23% or above. Similarly, about 30% of those holding STEM terminal master’s degrees, and 25% of those holding STEM terminal bachelor’s degrees, also scored in the top 4% of spatial ability (Wai et al., 2009).

Another study (Kell, Lubinski, Benbow, & Steiger, 2013) examined the spatial ability data for 563 participants from the Study of Mathematically Precocious Youth (SMPY; Shea et al., 2001). Levels of spatial ability, measured at age 13–14, added explanatory power 35 years later, accounting for 7.6% of the variance in creative achievement (number of patents and published articles), in addition to the 10.8% of variance explained by scores on the mathematics and verbal sections of the Scholastic Assessment Test (SAT). Lubinsky and team emphasized the necessity of adding a spatial assessment to talent search programs. This might help children and adolescents with high levels of spatial ability to reach their full potential. Without formal identification, spatially gifted adolescents may lack opportunities to develop their skills (Lohman, 1994; Lubinski, 2016), and even disengage from education (Lakin & Wai, 2020).

Despite being a robust predictor of future STEM achievement, spatial ability assessment is often not included in talent searches. This is because time for such assessments is generally limited and focused mostly on the numerical and verbal domains (Lakin & Wai, 2020). Few studies have examined the role of spatial ability in high achievement in nonacademic domains, such as sports and the arts. The results of existing studies are inconsistent, with some finding such links (Blazhenkova & Kozhevnikov, 2010; Hetland, 2000; Ivantchev, & Petrova, 2016; Jansen, Ellinger, & Lehmann, 2018, Notarnicola et al., 2014; Ozel, Larue, & Molinaro, 2002, 2004; Stoyanova, Strong & Mast, 2018), and others failing to do so (Chan, 2007; Heppe, Kohler, Fleddermann, & Zentgraf, 2016; Sala & Gobet, 2017). One way to improve understanding of the role of SA in high achievement is to use the same test battery in samples selected for high achievement in different domains. To our knowledge, our study is the first to carry out such an investigation.

Irrespective of achievement domain, it is not clear which spatial abilities are most relevant. Numerous spatial ability tests are available which tap into supposedly different processes, such as spatial information processing, mental rotation, spatial visualization, or manipulation of 2D and 3D objects (Uttal, Meadow, Tipton, Hand, Alden, & Warren, 2013).

However, several recent studies (Esipenko et al., 2018; Likhanov et al., 2018; Malanchini et al., 2019; Rimfeld et al, 2017) showed that spatial ability might have a unifactorial rather than multidimensional structure. For example, research has shown that the 10 spatial ability tests which form a King’s Challenge test battery (Rimfeld et al., 2017), constitute a single factor in British and Russian samples, explaining 42 and 40 percent of overall variance in spatial ability measures, respectively (Likhanov et al., 2018; Rimfeld et al., 2017). Interestingly, in a Chinese sample assessed with the same battery, a two-factorial structure of spatial ability emerged (explaining 40% of the total variance), with Cross-sections and Mechanical Reasoning forming a separate factor. Further research is needed to identify the sources of these differences across the samples.

The unifactorial structure of spatial ability was further demonstrated in another study that examined 16 measures of spatial ability in a UK sample (Malanchini et al., 2019). In this study, three factors emerged: navigation, object manipulation, and visualization; these in turn loaded strongly on a general factor of spatial ability. The unifactorial structure found in the UK and Russian samples suggests that, at least in these populations, a smaller number of tests can be used for rapid assessment of spatial ability.

The main purpose of the current study was to identify the most suitable spatial ability tests for creating a short online battery for educational assessment and talent identification. To this end, we investigated the psychometric properties of 10 spatial ability tests, as well as performance on these tests, in three adolescent samples selected for high achievement in science, arts, or sports. Comparison between these areas of expertise may provide additional insight into the role of spatial ability in these areas.

As the study was largely exploratory, we investigated the following research questions rather than testing specific hypotheses:

Research question 1: What are the best performing spatial ability tests in terms of psychometric properties?

Research question 2: What is the relationship between spatial ability and the three areas of expertise: Science, Sports, and Arts?

Research question 3: Does the previously shown unifactorial structure of spatial ability replicate in these expert samples?

## Method

### Participants

The study included 1470 adolescents, who were recruited at the Sirius educational center in Russia (645 males, 468 females, and 357 participants who did not provide information on gender). The ages of the participants ranged from 13 to 17 years (M = 14.78, SD = 1.20). Sirius is an educational center which provides intensive four-week educational programs for schoolchildren who have demonstrated high achievement in Science, Arts, or Sports. Adolescents from all regions of Russia are invited to apply for participation in these educational programs. Participation, as well as travel and other expenses, are free for participants. The socio-economic status (SES) of the participants was not measured. However, the participants likely represented a wide range of SES backgrounds, since the program application is open for everyone, participants come from all Russian geographic regions, and participation is fully funded.

We invited high-achievers to participate in one of the three tracks, selected on the basis of the following criteria:

– Science (339 males, 208 females): high school achievement, such as winning in a subject Olympiad (maths, chemistry, physics, informatics, IT, biology, etc.); or excellent performance in a scientific project;
– Arts (50 males, 198 females): winning in different competitions and demonstrating high achievement in painting, sculpture, choreography, literature, or music;
– Sports (220 males, 55 females): participation and winning in high-rank sport competitions (hockey, chess, and figure skating).

Due to the limited sample size, we were not able to analyze differences within the tracks (*e.g.,* math vs. chemistry; sculpture vs. choreography; or chess vs. hockey). We plan to explore those differences once the sample size needed for such research is achieved.

### Procedure

The study was approved by the Ethical Committee for Interdisciplinary Research. Parents or legal guardians of participants provided written informed consent. Additionally, verbal consent was obtained from the participants before the study. The testing took place in the regular classrooms of the educational center, which are quite similar to each other.

### Measures

#### King’s Challenge battery

Participants were presented with a gamified online battery called the “King’s Challenge” (KC), which had a test-retest reliability of r = 0.65 on average for the 10 spatial tests (Rimfeld et al., 2017); the battery was adapted for administration in Russian. The battery consists of 10 tests (see *Table 1*) and is gamified, with a general theme of building a castle and defending it against enemies. When they finished the battery, participants received feedback on their performance.

**Table 1:** Description of the 10 tests in the King’s Challenge battery

Subtest name	N of items	Time per item limit (sec)	Description
Cross-sections	15	20	visualizing cross-sections of objects
2D drawing	5	45	sketching a 2D layout of a 3D object from a specified viewpoint
Pattern assembly	15	20	visually combining pieces of objects to make a whole figure
Elithorn mazes	10	7	joining together as many dots as possible from an array
Mechanical reasoning	16	25	multiple-choice naive physics questions
Paper folding	15	20	visualizing placement of holes, after they punched through folded piece of paper
3D drawing	7	70	sketching a 3D drawing from a 2D diagram
Shape rotation	15	20	choosing the rotated target figure among others
Perspective-taking	15	20	visualizing objects from a different perspective
Mazes	10	25	searching for a way through a 2D maze in a time-limited task

*Note: Example items for each test are provided in the Supplementary Materials provided at the conclusion of this article. You will find the figures included there referenced with the S prefix in the text. Detailed information on the battery can be found in Rimfeld et al., 2017*.

We used the total of all correct items to score each test for use in further analysis. A total score for all 10 tests was computed by summing up the scores for each (KC Total), following the procedure described by Rimfeld and colleagues (2017).

#### Non-verbal intelligence

Non-verbal intelligence was measured by a shortened version of the Raven’s progressive matrices test (Raven, Raven, & Court, 1998). The test was modified to included six (only odd) items from the C, D, and E series, and three items from the F series (The A and B series were excluded). A discontinuation rule was applied in order to reduce the duration of the test: a series was terminated after three incorrect responses, and the test automatically progressed to the next series (in the F series, the test terminated immediately). The percentage of all correct responses out of the total number of 21 items was used for analysis.

#### Academic achievement

We used self-reported school Year grades for Math (Year grade Math) and the Russian Language (Year grade Rus). These grades are awarded by teachers to assess a student’s performance for the whole school year in a respective subject (based on performance across the year). The grading system is 1 to 5, where 1 = “terrible/fail”; 2 = “bad/fail”; 3 = “satisfactory”; 4 = “good”; and 5 = “excellent”. A 1 is practically never given, and a 2 is given only rarely (see Likhanov et al., 2020, for a discussion of the limitations of this grading system). In our sample, we had a restricted range of Year grades, with no 1 and 2 grades, since students who received these marks are unlikely to be invited to Sirius. The data for Year grades was available for 1109 participants.

We also collected self-reported grades for the State Final Assessment, a standardized exam hereafter referred to as the Exam. This test, taken at the end of 9th grade (15–16 years of age), is a measurement of students’ performance that serves as a major educational assessment tool. In the current study, only scores for the Math (Exam Math) and Russian language (Exam Rus) exams were used. Exam marks range from 1 to 5. No participants in our study had a 1 or 2 on this exam. The data for Exam results was available for only 306 participants, since not all study participants were of the age to undergo this exam at the time of data collection.

#### Spatial test selection criteria

In order to select the most informative spatial tests for educational assessment and talent search, we focused on six characteristics:

1. Absence of floor and ceiling effects — clustering of participants’ scores towards the worst or best possible scores (reflecting the unsuitability of the test difficulty level for the sample);
2. Differentiating power — the ability of the test to differentiate between Science, Arts, and Sports tracks in terms of average performance and distribution;
3. Low redundancy — this criterion allowed us to exclude tests which demonstrated very high correlations (above .7) with other tests in the battery;
4. Specificity — identifying tests that had small factor loadings on the latent “spatial ability” factor and/or loading on an additional factor, potentially suggesting specificity;
5. High reliability — having sufficiently high (.8) internal consistency;
6. High external validity — having common variance with non-verbal intelligence and educational achievement measures.

To check for floor and ceiling effects, we examined descriptive statistics, the shapes of distributions, and percentages of the highest and lowest values in each test. Distribution shapes also provided information on track differences. Differentiating power was further assessed with a series of ANOVAs. Factor structure was investigated by Principal Component Analysis (PCA). We also explored intercorrelations among all spatial measures to identify redundant tests indicated by strong bivariate correlations. Internal consistency was measured by the split-half reliability test, which randomly divides the test items into halves several times and compares the correlations between the two halves. External validity was assessed by correlating SA test scores with measures of non-verbal intelligence and academic achievement in Math and the Russian language.

Outliers were not deleted from the dataset, as we expect a significant proportion of children in this sample to demonstrate high performance in SA. For example, some studies showed that adolescents selected for math ability score higher than the third quartile of distribution in SA tests (see Benbow 1992; Lubinski & Dawis, 1992 for discussion), which is usually recognized as a threshold for outliers (Tukey, 1977). Similarly, some participants from non-academic tracks might show particularly low scores since they were not selected for the program based on academic achievement, or due to their investment of effort in sport or music training. For this reason, low outliers were also kept in the data set. The percentage of outliers ranged from 0.5 to 8.6% of the sample. Data on the number of outliers are presented in *Table S10*. (See Supplemental Materials)

Most of the analysis was done in SPSS 22.0. R 3.1 was used to clean the data, to calculate split-half reliability analysis and to draw correlation heatmaps.

## Results

### Data Analysis

**Figure S1.**
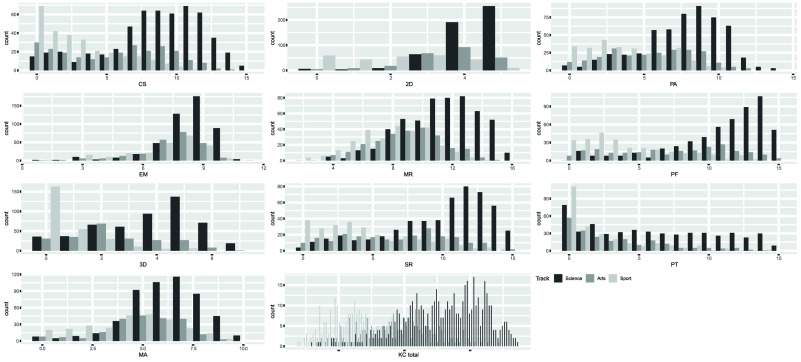
Distribution plots for three tracks: Science, Art, Sport.

Note: CS = Cross-sections; 2D = 2D-drawing; PA = Pattern assembly; EM = Elithorn mazes; MR = Mechanical reasoning; PF = Paper folding; 3D = 3D-drawing; SR = Shape rotation; PT = Perspective-taking; MA = Mazes; KC Total = total score for King’s Challenge battery.

The main purpose of the current study was to identify the most suitable spatial ability tests for creation of a short online battery for educational assessment and talent identification. Specifically, we examined six test characteristics as described in the method section. Descriptive statistics for the whole sample and for different tracks separately are presented in *Tables 2* and *3*. *Figure S1* (See Supplemental Materials) presents distributions for all tests for each track. The numbers differed for different measurements: for spatial ability measurements, the missing data ranged from 52 to 264, as some participants did not complete the whole battery; for Year grades, the missing data ranged from 359 to 402, as these participants did not report their grades. In addition, as explained above, the data for Exams was available only for the older subsample which had completed the Exam. In most analyses reported in this paper, we used the data for the maximum number of participants which was available for each measure.

**Table 2:** Descriptive statistics for the whole sample: number of correct responses in spatial ability measures, exam and year grades, and non-verbal intelligence

Test (number of items)	N	Mean (SD)	Min	Max	Skewness
Cross-sections (15)	1418	6.11 (4.16)	0	15	0.026
2D drawing (5)	1356	3.38 (1.45)	0	5	–0.912
Pattern assembly (15)	1414	6.00 (3.31)	0	14	–0.125
Elithorn mazes (10)	1206	7.77 (1.68)	0	10	–1.239
Mechanical reasoning (16)	1412	9.80 (2.92)	2	16	–0.137
Paper folding (15)	1404	8.06 (4.71)	0	15	–0.226
3D drawing (7)	1351	2.50 (2.03)	0	6.9	0.340
Shape rotation (15)	1373	7.30 (4.42)	0	15	–0.077
Perspective–taking (15)	1360	4.24 (4.28)	0	15	0.819
Mazes (10)	1357	5.31 (2.20)	0	10	–0.486
KC total (123)	1356	60.62 (23.65)	11.5	111.6	0.080
Exam Math (2-5)	306^	4.79 (0.53)	3	5	–2.29
Exam Rus (2-5)	306^	4.83 (0.49)	3	5	–2.56
Year grade Math (2-5)	1068	1.00 (0.72)	3	5	–0.63
Year grade Rus (2-5)	1111	4.44 (0.63)	3	5	–1.01
Raven’s score (21)	1327	0.74 (0.17)*	0.05	1	–0.9

Note. Total = total score for King’s Challenge battery; the number of items in each test is presented in brackets; * Raven’s score is calculated by dividing the number of correct answers by the total number of items; ^ The N for Exam was low because most of the study participants had not reached the age when this Exam is taken.

**Table 3:** Descriptive statistics for all Tracks: spatial ability, exam performance, and non-verbal intelligence

	Science				Art				Sport			
Test (number of items)	N	(Mean SD)	Min	Max	N	(Mean SD)	Min	Max	N	Mean (SD)	Min	Max
Cross-sections (15)	547	8.61 (3.57)	0	15	248	5.62 (3.77)	0	14	275	2.88 (2.76)	0	11
2D drawing (5)	529	4.22 (.86)	0	5	243	3.57 (1.08)	0	5	270	2.05 (1.43)	0	4.9
Pattern assembly (15)	546	7.85 (2.75)	0	14	248	5.52 (2.99)	0	12	274	3.71 (2.66)	0	10
Elithorn mazes (10)	488	8.34 (1.51)	0	10	238	7.40 (1.56)	1	10	234	7.05 (1.85)	0	10
Mechanical reasoning (16)	546	11.43 (2.53)	4	16	246	9.06 (2.36)	4	15	274	7.86 (2.42)	2	14
Paper folding (15)	545	11.11 (3.49)	1	15	239	7.43 (4.20)	0	15	274	4.03 (3.33)	0	13
3D drawing (7)	521	3.78 (1.81)	0	6.91	229	2.45 (1.62)	0	6.77	270	.78 (1.07)	0	5.1
Shape rotation (15)	532	9.99 (3.70)	0	15	226	6.61 (3.78)	0	15	269	4.34 (3.52)	0	14
Perspective–taking (15)	527	6.17 (4.63)	0	15	220	3.34 (3.44)	0	14	268	2.32 (3.09)	0	14
Mazes (10)	526	6.28 (1.90)	0	10	218	5.06 (1.94)	0	9	268	4.17 (2.27)	0	9
KC total (123)	526	78.2 (18.2)	18.2	111.6	218	56.1 (16.9)	19.7	103.1	267	39.1 (14.6)	11.5	87.5
Exam Math (2–5)	203	4.93 (.43)	4	5	93	4.57 (.60)	3	5	10	3.90 (.32)	3	4
Exam Rus (2–5)	203	4.86 (.50)	3	5	93	4.80 (.46)	3	5	10	4.50 (.53)	4	5
Year Math grade (2–5)	537	4.79 (.50)	3	5	249	4.45 (.78)	3	5	282	3.95 (.71)	3	5
Year grade Rus (2–5)	554	4.58 (.59)	3	5	254	4.65 (.51)	3	5	303	4.02 (.58)	3	5
Raven’s score (21)	504	.83 (.12)*	.14	1	220	.73 (.15)*	.24	1	259	.60 (.18)*	.05	1

Note. The number of items (possible range) is shown in brackets next to each test name with the name of the subtest. KC Total = total score for King’s Challenge battery; the number of items in each test is pre-sented in the brackets; * Raven’s score is calculated by dividing the number of correct answers by the total number of items; ^Total score for 2D and 3D drawing tasks had decimals as a score for an individ-ual trial in both tests ranged from 0 to 1, reflecting the number of correct lines drawn in the time given for this trial.

#### Absence of floor and ceiling effects

Mechanical reasoning and Mazes demonstrated normal distribution, both across and within tracks. For Shape rotation, Paper folding, and Pattern assembly, the scores were negatively skewed for the Science track and positively skewed for the Sports tracks. Shape rotation, Paper folding, and Cross-sections tests demonstrated bimodal distributions for the whole sample. The ceiling effect for the whole sample was observed for the 2D-drawing and Elithorn mazes tests: in the 2D-drawing test, 43% of participants had scores of 4 or 5 (out of 5); in the Elithorn mazes test, 53% of participants had scores from 8 to 10 (out of 10). The floor effect was present in 3D-drawing and Perspective-taking tests: for the 3D-drawing test, 46.9% of participants had scores of 2 or lower (out of 7), and for Perspective-taking test, 54% of participants had scores of 3 or lower (out of 15).

For further investigation of the floor and ceiling effects, we estimated the dif-ficulty of each test by calculating the percentages of correct responses (see *Table S1*). For the whole sample, the Elithorn mazes and 2D-drawing were the easiest tests in the battery (77.7% and 68% of responses correct, respectively), whereas Perspective-taking was the most difficult one (28.2% responses correct).

#### Differentiating power

We used ANOVA to examine potential differences among the Science, Arts, and Sports tracks. As described in the Method section, gender distribution across tracks was uneven. Previous studies that employed the same SA battery showed moderate gender differences in a British sample of young adults (Toivainen et al, 2018) and samples of Russian (Esipenko et al., 2018) and Chinese students (Likhanov et al., 2018). We examined gender effects in 11 one-way ANOVAs (10 tests and the total score) that showed male advantage for three tests, as well as a total SA score, and female advantage for two tests. All effects were negligible to modest (between .004 and .05; See *Table S2* for details). Gender was regressed out in all further analyses.

Thereafter, these standardized residuals were used in one-way ANOVAs to compare educational tracks (Science, Arts, and Sports). Homogeneity of variance was assessed by the Levene’s test (Levene, 1960). Welch’s ANOVA was used to account for the heterogeneity of variance in some tests (Field, 2013). Variance heterogeneity among tracks was found for all tests (p ≤ 0.01), with the exception of Mechanical reasoning (p = 0.25) and Shape rotation (p = 0.13).

**Figure 1.**
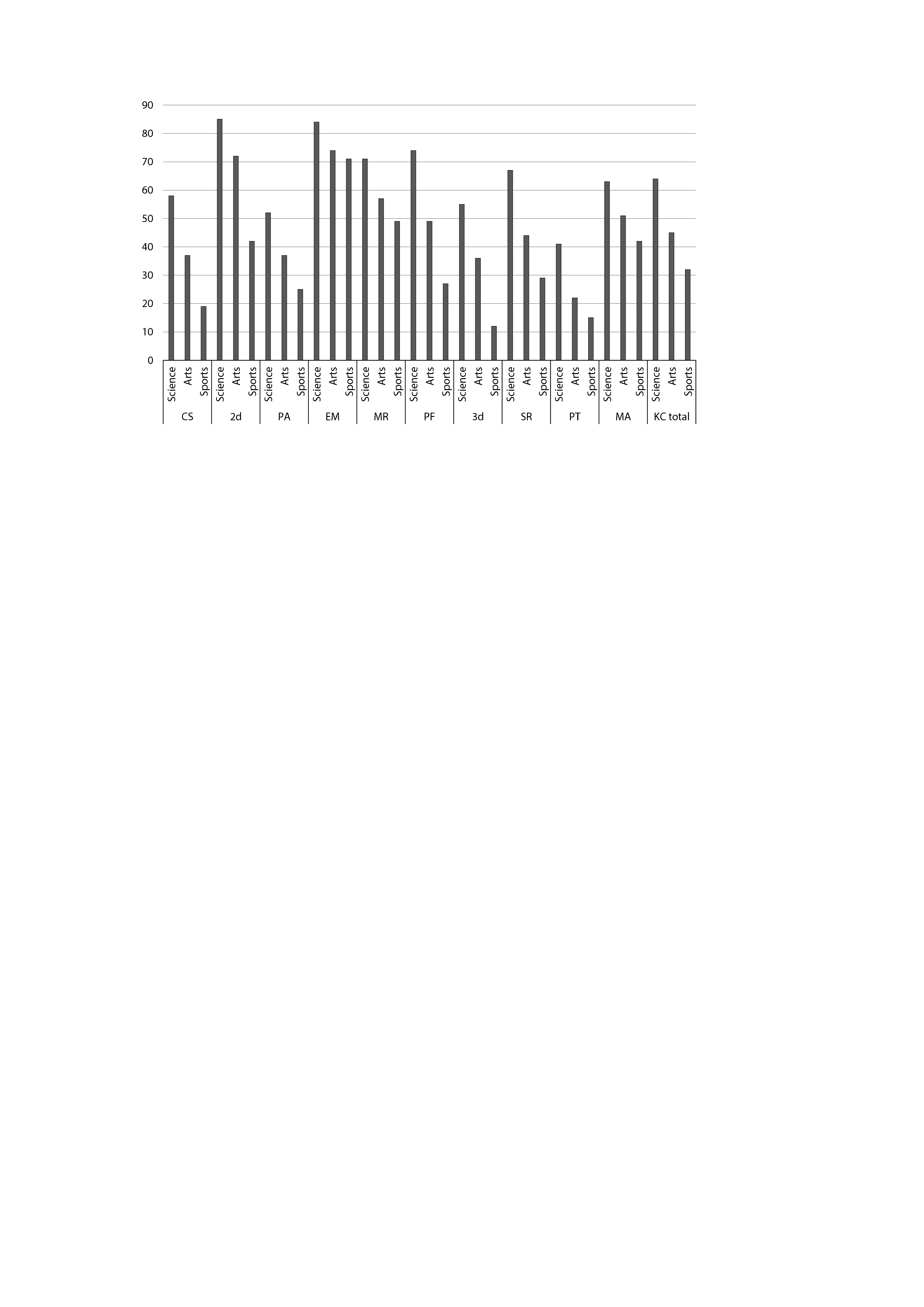
Percent of correct scores for each test across the three tracks.

Overall, the ANOVAs showed significant average differences across the three tracks in every spatial measure and the total score, with effect sizes (η^2^) ranging from .13 to .65. The results of Welch’s F-tests, p-values, and η^2^are presented in *Table S3*. Due to non-normal distribution within tracks in all tests, with the exception of Mechanical reasoning and Mazes, we conducted non-parametric tests to confirm the results of the ANOVA. The Kruskal-Wallis H test confirmed significant differences between tracks in all spatial tests and total scores (χ^2^ (3, N = 1070) = [133.1 – 423.5]; p < .01). Means for all SA tests according to track are presented in *Figure 1*. Post-hoc analyses showed that each track significantly differed from each other track in each test (p < .05 for all comparisons). The science track had the highest scores and the Sports track had the lowest.

CS = Cross-sections; 2D = 2D-drawing; PA = Pattern assembly; EM = Elithorn mazes; MR = Mechanical reasoning; PF = Paper folding; 3D = 3D-drawing; SR = Shape rotation; PT = Perspective-taking; MA = Mazes; KC Total = total score for King’s Challenge battery.

Significant differences across the tracks were also found for non-verbal intelligence (F (2, 980) = 19.42; p < .01; η^2^= .31), with means of .83 (SD = .12), .73 (SD = .15), and .60 (SD = .18) for the Science, Arts, and Sports tracks, respectively.

**Table 4:** Correlational matrix for the whole sample (N = 1150-1412; p<0.05 for all correlations)

	1	2	3	4	5	6	7	8	9	10	KC total
1. CS	1	.582^**^	.506^**^	.339^**^	.535^**^	.617^**^	.630^**^	.527^**^	.431^**^	.384^**^	.768^**^
2. 2d	.582^**^	1	.579^**^	.390^**^	.584^**^	.672^**^	.673^**^	.580^**^	.476^**^	.474^**^	.779^**^
3. PA	.506^**^	.579^**^	1	.358^**^	.523^**^	.597^**^	.600^**^	.551^**^	.405^**^	.415^**^	.746^**^
4. EM	.339^**^	.390^**^	.358^**^	1	.453^**^	.389^**^	.428^**^	.392^**^	.340^**^	.368^**^	.552^**^
5. MR	.535^**^	.584^**^	.523^**^	.453^**^	1	.609^**^	.591^**^	.547^**^	.505^**^	.466^**^	.774^**^
6. PF	.617^**^	.672^**^	.597^**^	.389^**^	.609^**^	1	.712^**^	.623^**^	.459^**^	.497^**^	.848^**^
7. 3d	.630^**^	.673^**^	.600^**^	.428^**^	.591^**^	.712^**^	1	.652^**^	.529^**^	.531^**^	.838^**^
8. SR	.527^**^	.580^**^	.551^**^	.392^**^	.547^**^	.623^**^	.652^**^	1	.462^**^	.492^**^	.799^**^
9. PT	.431^**^	.476^**^	.405^**^	.340^**^	.505^**^	.459^**^	.529^**^	.462^**^	1	.382^**^	.689^**^
10. MA	.384^**^	.474^**^	.415^**^	.368^**^	.466^**^	.497^**^	.531^**^	.492^**^	.382^**^	1	.638^**^
KC total	.768^**^	.779^**^	.746^**^	.552^**^	.774^**^	.848^**^	.838^**^	.799^**^	.689^**^	.638^**^	1

*CS = Cross-sections; 2D = 2D-drawing; PA = Pattern assembly; EM = Elithorn mazes; MR = Mechanical reasoning; PF = Paper folding; 3D = 3D-drawing; SR = Shape rotation; PT = Perspective-taking; MA = Mazes; KC Total = total score for King’s Challenge battery*.

#### Low Redundancy

All pairwise correlations were significant and positive, ranging from r = .34 to r = .85 (*Tables S4* for within-track correlations). The data showed the highest correlations for the 3D-drawing, 2D-drawing, and Paper folding tests (>.67), which suggests that having all of them in one battery is unnecessary. Elithorn mazes and Mazes tests showed the lowest correlations with other spatial ability tests within the Arts track and the whole sample.

#### Specificity

We performed Principal Component Analysis (PCA) on the raw data (sum of the correct responses for each spatial test) for the whole sample and individual tracks. To ensure that the data was suitable for factor analysis, we applied the Kaiser-Meyer-Olkin (KMO) measure of sampling adequacy and the Bartlett’s test of sphericity for both the whole sample and each track separately (see *Table S5*). The results indicated that the data was suitable for factor analysis (Hair et al., 1998).

For the whole sample, the PCA scree plot (see *Figure S2*) and the eigenvalues suggested single factor extraction (explaining 56.48% of variance; see *Table 5*). All tests showed high loadings on this factor (.58 – .85). For the Science and Sports tracks, the factor structure was also unifactorial: a single factor explained 45.76% and 38.74% of variance, respectively. For the Arts track, two factors explained 50.41% of variance: factor 1 = 39.68%; and factor 2 = 10.79%. Factor 1 included all tests except the Elithorn mazes and Mazes, which formed factor 2. These findings indicate that one test from a battery would be able to assess the underlying spatial ability factor to some degree. Factor loadings and eigenvalues for the whole sample and each track separately are shown in *Table 5*.

**Table 5:** Factor analysis results: component matrices for the whole sample and each track separately

Test	Whole sample N=1086	Science N=443	Arts N=203		Sports N=223
	Component	Component	Component		Component
	1	1	1	2	1
Cross-sections	0.75	0.64	0.71		0.4
2D drawing	0.81	0.67	0.71		0.77
Pattern assembly	0.74	0.63	0.71		0.5
Elithorn mazes	0.58	0.56		0.53	0.55
Mechanical reasoning	0.79	0.69	0.62		0.7
Paper folding	0.84	0.76	0.69		0.64
3D drawing	0.85	0.79	0.67		0.74
Shape rotation	0.79	0.69	0.65		0.6
Perspective-taking	0.66	0.66	0.51		0.57
Mazes	0.66	0.62		0.85	0.67
Eigenvalues	5.65	4.58	3.96	1.07	3.87
% of variance explained	56.48	45.76	39.68	10.79	45.76

#### Reliability

Split-half reliabilities for the whole sample and separate tracks are shown in *Table S6*. Split-half reliability varied from weak to strong across the tests in the whole sample (r = .27 – .95). High reliabilities (> .8) were shown for Cross-sections, 2D drawing, Pattern assembly, Paper folding, 3D drawing, Shape rotation, and Perspective-taking. Moderate reliabilities were shown (>.65) for Mechanical reasoning and Mazes. Low reliability (.27) was shown for Elithorn mazes. The pattern of reliability was similar for all tracks.

#### External validity

*Table 6* presents the correlations between the spatial ability tests, Raven’s progressive matrices, and academic achievement for the full sample (see *Tables S7 — S9* for correlations within tracks).

**Table 6:** Correlations for spatial measures with non-verbal intelligence, and Year grades (whole sample)

Test	Nonverbal intelligence N=1327	Year grade Maths N = 907–1013	Year grade Rus N = 957–1166	Fisher’s Z Maths vs. Rus
Cross-sections	.49^**^	.38^**^	.21^**^	4.32^**^
2D drawing	.62^**^	.44^**^	.30^**^	4.19^**^
Pattern assembly	.51^**^	.38^**^	.22^**^	4.05 ^**^
Elithorn mazes	.40^**^	.24^**^	.16^**^	1.78
Mechanical reasoning	.53^**^	.37^**^	.16^**^	4.7^**^
Paper folding	.59^**^	.44^**^	.30^**^	4.62^**^
3D drawing	.59^**^	.44^**^	.28^**^	4.47^**^
Shape rotation	.53^**^	.35^**^	.22^**^	4.49^**^
Perspective-taking	.38^**^	.27^**^	.12^**^	3.9^**^
Mazes	.47^**^	.33^**^	.20^**^	3.42^**^
KC total	.68^**^	.49^**^	.29^**^	5.88^**^

Note.* p ≤ 0.05. ** p ≤ 0.001. Fisher’s Z refers to the comparison between correlations of spatial scores with Math vs. Russian grades.

All tests showed significant positive weak to strong correlations with non-verbal intelligence: *r* (1325) = [.38 – .62], *p* ≤ .01 for the whole sample and within tracks.

For the whole sample, SA was correlated with the Year grades for both Mathematics (*r*(1056) = [.24 – .49], *p* ≤ .01), and the Russian language, (*r* (1107) = [.12 – .30], *p* ≤ .01.) Fisher’s r-z transformation showed that correlations were higher for Math than for Russian (z = [3.9 – 5.88], *p* ≤ .01), with the exception of Elithorn mazes.

The pattern of correlations between the students’ Year grades and SA tests was slightly different within tracks (see *Table S10*). On the Science track, there were significant weak to moderate correlations between SA tests and Year grade for Mathematics (*r* (547) = [.12 – .30], *p* ≤ .01), but no correlations between spatial tests and the Year grade for the Russian language. On the Arts and Sports tracks, there were consistent significant correlations between the Year grades in Math and SA, and some between Year grades in Russian and SA (Fisher’s Z was non-significant).

*Tables S10* and *S11* present the results for correlations between SA and the Exam. In the whole sample, the Math Exam showed weak to moderate correlations with SA (*r*(304) = [.20 – .34], *p* ≤ .05); the Russian Exam was only weakly correlated with SA (*r*(304) = [.12 – .16], *p* ≤ .05). Within tracks, only a few correlations between SA and Exam reached significance.

#### Tests selected for inclusion in the Online Short Spatial Ability Battery ( OSSAB)

Four of the tests matched the criteria for selection, including the predicted pattern of moderate correlations with nonverbal intelligence and mathematics achievement (*e.g*., Tosto et al., 2014). Below we describe the selected tests:

1. *Paper Folding* is a widely used measure of spatial visualization (Carrol, 1993), which has previously been recommended for talent identification (Hegarty & Waller, 2005; Linn & Petersen, 1985; Uttal et al., 2012). In the present study, *Paper Folding* appeared very similar to 2D and 3D drawing tests in correlational patterns, discriminant validity, factor loadings, and reliability. However, 2D and 3D drawing tests were excluded, as they showed either ceiling or floor effects;
2. *Shape Rotation* taps into a different dimension of spatial ability — mental rotation (Shepard & Metzler, 1971). This parameter was selected as it matched all established criteria, including high reliability and different distributions for the different tracks;
3. *Mechanical Reasoning* taps into a construct of Mechanical Aptitude — the ability to understand and apply mechanical concepts and principles to solve problems (Wiesen, 2015); it is recognized as important in educational tracking and career planning (Muchinsky, 1993). We selected the *Mechanical Reasoning* test, which showed better results than Cross-sections and Elithorn mazes in terms of normally distributed scores for all three tracks, as well as significant track differences;
4. *Pattern assembly* measures spatial relations — another important aspect of spatial ability (Carrol, 1993). This test showed the same pattern of distribution across tracks (along with Shape Rotation and Paper Folding), as well as high reliability, high factor loadings, and good correlations with other tests. By contrast, Mazes had low correlations with other tests and low discriminant validity; and Perspective-taking had high reliability, factor loadings, and correlations with other tests, but showed a strong floor effect.

## Discussion

The purpose of the present study was to investigate the psychometric properties and factor structure of 10 spatial ability tests in order to create a short battery suitable for educational assessment and talent search. We collected data using an existing extensive spatial ability battery (King’s Challenge; Rimfeld et al., 2017) in a sample of schoolchildren who had demonstrated high achievement in Science, Arts, or Sports. Based on our analysis, four tests were identified to be included into an Online Short Spatial Ability Battery “OSSAB.” The following four best-performing tests were selected: Paper Folding, Shape Rotation, Mechanical Reasoning, and Pattern Assembly. All selected tests are available at https://github.com/fmhoeger/OSSAB.

We analyzed our data to demonstrate the utility of the OSSAB for educational purposes. In particular, we ran the analysis by splitting the sample into three educational tracks (Science, Arts, and Sports). The analysis showed significant differences between tracks, with η^2^ranging from .32 to .67. For example, the Science track showed the highest results in all four tests. We also compared the results of the Science track with previous results and found higher average performance in the Science track than that of unselected university students from China and Russia (Esipenko et al., 2018; Likhanov et al., 2018) and of an unselected population of young adults from UK (Rimfeld et al., 2017). Our result was also consistent with repeatedly found correlations between math and spatial ability (.43; Tosto et al., 2014), and between intelligence and academic achievement (.60 - .96; Bouchard & Fox, 1984; Deary, Strand, Smith, & Fernandes, 2007; Kemp, 1955; Wiseman, Meade, & Parks, 1966). Considering that SA was not part of the admission criteria for the Science track, the results suggest that SA might be a useful marker for high STEM performance.

These results provide further support for adding SA tests to verbal and math tests in order to establish patterns of strengths and weaknesses that can be predictive of future achievement in different domains (Shea, Lubinski, & Benbow, 2001; Webb, Lubinski, & Benbow, 2007). Moreover, talent search programs that focus mostly on verbal and math ability may overlook people with high SA only, which may lead to disengagement and behavioral problems in these young people (Lakin & Wai, 2020). These individuals will benefit from early identification of their high SA, and from personalized educational programs that capitalize on their strengths, including such activities as electronics, robotics, and mechanics.

For the Sports track, a positive skew was shown in Shape rotation, Paper folding, and Pattern assembly. It is possible that the relatively low performance of the Sports track on SA and other cognitive and academic achievement measurements is the result of these students’ extreme investment of effort in sports training (see Likhanov, 2021, in preparation; for discussion). It is also common for athletes to disengage from traditional academic studies (Adler & Adler, 1985) and fall behind academically (*e.g.,* due to attending training camps). SA training that involves more enjoyable activities — for example, using computer games and VR or AR (augmented reality) (Uttal et al., 2014, Papakostas et al., 2021) — might be beneficial for their academic performance.

It is also possible that the battery used in this study did not tap into the ability of athletes to process visuo-spatial information in a natural environment, such as attentional processes or long-term working memory, which was shown in some studies to be highly developed in professional athletes, including hockey players (Belling et al 2015; Mann et al, 2007; Voss et al., 2010). The tests in this study measured mostly small-scale SA, *i.e.,* the ability to mentally represent and transform two- and three-dimensional images that can typically be apprehended from a single vantage point (Likhanov et al., 2018; Wang and Carr, 2014). Further research is needed that includes both small- and large-scale spatial ability tests.

For the Arts track, the average performance fell somewhere in between the Science and Sports tracks. This track is heterogeneous, but the sample size was not large enough to investigate spatial ability in separate sub-tracks (*e.g.,* fine arts vs. music). Therefore, in this study, the Arts track can be considered unselected in terms of academic achievement.

Cross-track differences also emerged in the structure of SA. Results from the factor analysis for the whole sample on the Science and Sports tracks replicated the previous findings of the unifactorial structure of the spatial ability (Esipenko et al., 2018; Likhanov et al., 2018; Rimfeld et al., 2017). However, for the Arts track, a two-factorial structure emerged (Elithorn mazes and Mazes tests formed the second factor).

A number of speculative explanations for this can be proposed. The Arts track included high achievers in music (20%), literature (40%), and fine art (30%). The second factor may reflect an advanced ability of the fine art students to process visual information in two-dimensional space, as these two tests are hypothesized to measure an ability for 2D image scanning (Poltrock, & Brown, 1984). Alternatively, a number of methodological issues may also have led to the second factor on the Arts track. The two tests showed lower correlations with other spatial ability measures (lower than .26) for the Arts track, which could have stemmed from the smaller sample size for this track (though sufficient, *e.g*., according to Comrey and Lee, 1992) and lower reliability of the two tests.

## Conclusion

The *Online Short Spatial Ability Battery (OSSAB)* can be used for talent identification, educational assessment, and support. Future research is needed to evaluate the use of this battery with other specific samples and unselected populations.

## Limitations

Our study had a number of limitations. Firstly, sample sizes differed among sex and track groups, precluding fine-grained investigation of these effects. Secondly, the study had only limited access to students’ academic achievement: the majority of the sample had not yet taken the state exam; and the Year grades only provided a very crude estimate of achievement as they range from 2 to 5, with 2 absent from this sample. Thirdly, as mentioned above, large-scale spatial ability was not measured in the current study. Further research is needed to evaluate the relative strengths and weaknesses in small- and large-scale spatial abilities for different tracks. Fourthly, there were some differences in reliability across measures. Moreover, some tests could be more enjoyable. Future research needs to explore whether and how enjoyment may be related to the test validity.

## Appendix

**Table S1:** Proportion (%) of correct responses for King’s Challenge tests.

Test	Whole sample	Science	Arts	Sports
	Mean* (SD)	Mean (SD)	Mean (SD)	Mean (SD)
Cross-sections	40.77 (27.72)	57.37 (23.77)	37.45 (25.15)	19.22 (18.38)
2D drawing	68.04 (28.65)	84.68 (17.03)	71.66 (21.50)	41.34 (28.55)
Pattern assembly	39.97 (22.09)	52.32 (18.35)	36.77 (19.94)	24.74 (17.72)
Elithorn mazes	77.72 (16.69)	83.64 (14.61)	73.92 (15.60)	71.20 (18.11)
Mechanical reasoning	61.38 (18.07)	71.45 (15.83)	56.61 (14.74)	49.13 (15.11)
Paperfolding	53.71 (31.38)	74.09 (23.30)	49.54 (28.03)	26.86 (22.20)
3D drawing	36.62 (29.00)	54.35 (25.75)	35.33 (23.21)	11.76 (15.86)
Shape rotation	48.64 (29.46)	66.58 (24.67)	44.04 (25.20)	28.95 (23.47)
Perspective - taking	28.25 (28.56)	41.15 (30.86)	22.27 (22.96)	15.45 (20.62)
Mazes	53.14 (21.97)	62.78 (19.02)	50.64 (19.40)	41.68 (22.75)
KC Total	49.26 (19.23)	63.56 (14.80)	45.59 (13.74)	31.80 (11.87)

*Note: *Proportion (%) of correct responses; the tests represent tests from King’s Challenge battery (Rimfeld et al., 2017); KC Total = total scoresfor King’s Challenge battery.*

**Table S2:** ANOVA for sex within track and the whole sample for all SA subtests

Test (number of items)	Sex	Whole sample					Science track					Arts track					Sports track				
		N	M	(SD)	F	η^2^	N	M	(SD)	F	η^2^	N	M	(SD)	F	η^2^	N	M	(SD)	F	η^2^
Cross-sections (15)	male	609	6.45	(4.37)	10.6	ns	339	8.99	(3.52)	10.6**	0.02	50	5.32	(3.68)	0.39	ns	220	2.8	(2.67)	0.91	ns
	female	461	6.43	(3.94)			208	7.98	(3.55)			198	5.69	(3.8)			55	3.2	(3.09)		
2D drawing (5)	male	592	3.4	(1.57)	12.19*	0.01	327	4.32	(0.82)	12.2**	0.02	49	3.53	(1.3)	0.07	ns	216	1.96	(1.42)	4.36**	0.02
	female	450	3.65	(1.14)			202	4.06	(0.9)			194	3.57	(1.01)			54	2.41	(1.41)		
Pattern assembly (15)	male	607	6.34	(3.48)	20	ns	338	8.25	(2.68)	20**	0.04	50	5.04	(3.16)	1.59	ns	219	3.67	(2.68)	0.25	ns
	female	461	6.13	(3.02)			208	7.19	(2.75)			198	5.64	(2.94)			55	3.87	(2.58)		
Elithorn mazes (10)	male	534	7.97	(1.8)	27.59**	0.01	299	8.62	(1.47)	27.6**	0.05	50	7.55	(1.38)	0.57	ns	185	7.04	(1.93)	0.01	ns
	female	426	7.56	(1.57)			189	7.9	(1.47)			188	7.36	(1.6)			49	7.06	(1.54)		
Mechanical reasoning(16)	male	607	10.4	(3.16)	99.57**	0.03	338	12.2	(2.29)	99.6**	0.16	50	8.94	(2.73)	0.15	ns	219	7.86	(2.43)	0.02	ns
	female	459	9.43	(2.46)			208	10.2	(2.4)			196	9.09	(2.26)			55	7.85	(2.38)		
Paper folding (15)	male	607	8.18	(5.07)	7.05**	0.01	338	11.4	(3.49)	7.05**	0.01	50	5.98	(4.45)	7.74**	0.03	219	3.67	(3.21)	13.5**	0.05
	female	451	8.81	(4.13)			207	10.6	(3.45)			189	7.81	(4.06)			55	5.47	(3.45)		
3D drawing (7)	male	589	2.65	(2.24)	18.9	ns	324	4.04	(1.86)	18.9**	0.04	48	2.01	(1.67)	4.52**	0.02	217	0.71	(1.05)	5.31**	0.02
	female	431	2.74	(1.73)			197	3.34	(1.66)			181	2.56	(1.59)			53	1.09	(1.11)		
Shape rotation (15)	male	602	7.94	(4.64)	18.9	ns	335	10.5	(3.62)	18.9**	0.04	49	6.27	(3.68)	0.51	ns	218	4.36	(3.56)	0.04	ns
	female	425	7.52	(4.04)			197	9.09	(3.67)			177	6.7	(3.81)			51	4.25	(3.38)		
Perspective – taking (15)	male	598	5.37	(4.72)	82.94**	0.05	332	7.48	(4.63)	82.9**	0.14	49	3.8	(3.49)	1.1	ns	217	2.5	(3.22)	3.93**	0.02
	female	417	3.35	(3.52)			195	3.94	(3.69)			171	3.21	(3.43)			51	1.55	(2.38)		
Mazes (10)	male	596	5.47	(2.33)	9.41	ns	331	6.47	(1.83)	9.41**	0.02	48	4.9	(1.99)	0.46	ns	217	4.06	(2.32)	2.59	ns
	female	416	5.44	(2.02)			195	5.95	(1.97)			170	5.11	(1.93)			51	4.63	(2.05)		
KC total (123)	male	595	64.4	(26.7)	57.68**	0.00	331	82.6	(17.7)	57.7**	0.1	48	53.8	(18.2)	1.1	ns	216	38.8	(14.6)	0.79	ns
	female	416	61.3	(19.1)			195	70.7	(16.5)			170	56.7	(16.5)			51	40.8	(14.5)		

*Note: ** — p%0.001; KC Total = total score for King’s Challenge battery, ns — nonsignificant*

**Table S3:** ANOVA for the three Tracks

Test	Track	N	M (SD)	Levene’s p-value test	F	η^2^
Cross-sections	Science	547	8.61 (3.56)			
	Arts	248	5.62 (3.77)	.00	322.96^**^	0.495
	Sports	275	2.88 (2.75)			
2D drawing	Science	529	4.22 (.86)			
	Arts	243	3.57 (1.08)	.00	265.17^**^	0.648
	Sports	270	2.05 (1.43)			
Pattern assembly	Science	546	7.85 (2.75)			
	Arts	248	5.52 (2.99)	.00	227.03^**^	0.402
	Sports	274	3.71 (2.65)			
Elithorn mazes	Science	488	8.34 (1.51)			
	Arts	238	7.4 (1.55)	.00	55.72^**^	0.128
	Sports	234	7.05 (1.85)			
Mechanical reasoning	Science	546	11.43(2.53)			
	Arts	246	9.06 (2.35)	.79	229.29^**^	0.413
	Sports	274	7.86 (2.41)			
Paper folding	Science	545	11.11(3.49)			
	Arts	239	7.43 (4.20)	.00	399.62^**^	0.671
	Sports	274	4.03 (3.33)			
3D drawing	Science	521	3.78 (1.81)			
	Arts	229	2.45 (1.62)	.00	424.10^**^	0.607
	Sports	270	0.78 (1.07)			
Shape rotation	Science	532	9.99 (3.7)			
	Arts	226	6.61 (3.78)	.00	229.45^**^	0.321
	Sports	269	4.34 (3.52)			
Perspective-taking	Science	527	6.17 (4.62)			
	Arts	220	3.34 (3.44)	.00	123.57^**^	0.448
	Sports	268	2.32 (3.09)			
Mazes	Science	526	6.28 (1.9)			
	Arts	218	5.06 (1.94)	.00	94.62^**^	0.203
	Sports	268	4.17 (2.27)			
KC total	Science	526	78.18(18.21)			
	Arts	218	56.08 (16.9)	.00	557.71^**^	0.492
	Sports	267	39.17(14.6)			

*Note: ** — p≤0.001; KC Total = total scores for King’s Challenge battery. Sex is regressed out from all scores for this analysis*.

**Table S4:** Bivariate correlations for the three tracks

***Science (N = 468 – 546)***										
Test	1	2	3	4	5	6	7	8	9	10
1 Cross-sections										
2 2D drawing	.42^**^									
3 Pattern assembly	.35^**^	.38^**^								
4 Elithorn mazes	.27^**^	.31^**^	.27^**^							
5 Mechanical reasoning	.42^**^	.40^**^	.42^**^	.37^**^						
6 Paper folding	.48^**^	.50^**^	.43^**^	.33^**^	.47^**^					
7 3D drawing	.49^**^	.49^**^	.45^**^	.40^**^	.45^**^	.56^**^				
8 Shape rotation	.35^**^	.42^**^	.43^**^	.32^**^	.42^**^	.48^**^	.50^**^			
9 Perspective-taking	.33^**^	.40^**^	.31^**^	.33^**^	.44^**^	.38^**^	.48^**^	.39^**^		
10 Mazes	.26^**^	.31^**^	.33^**^	.30^**^	.35^**^	.39^**^	.47^**^	.41^**^	.32^**^	
11 KC total	.67^**^	.62^**^	.64^**^	.53^**^	.70^**^	.75^**^	.76^**^	.73^**^	.70^**^	.58^**^
***Arts (N = 213 – 248)***										
Test	1	2	3	4	5	6	7	8	9	10
1 Cross-sections										
2 2D drawing	.43^**^									
3 Pattern assembly	.33^**^	.47^**^								
4 Elithorn mazes	.15^*^	.16^*^	.22^**^							
5 Mechanical reasoning	.35^**^	.38^**^	.35^**^	.32^**^						
6 Paper folding	.40^**^	.41^**^	.44^**^	.18^**^	.50^**^					
73D drawing	.37^**^	.54^**^	.41^**^	.24^**^	.39^**^	.47^**^				
8 Shape rotation	.36^**^	.39^**^	.45^**^	.26^**^	.36^**^	.44^**^	.48^**^			
9 Perspective-taking	.22^**^	.25^**^	.30^**^	.16^*^	.24^**^	.29^**^	.29^**^	.34^**^		
10 Mazes	-.03	.16^*^	.11	.14^*^	.15^*^	.22^**^	.24^**^	.23^**^	.05	
11 KC total	.63^**^	.62^**^	.68^**^	.41^**^	.66^**^	.76^**^	.68^**^	.74^**^	.55^**^	.32^**^
***Sports (N = 234 – 275)***										
Test	1	2	3	4	5	6	7	8	9	10
1 Cross-sections										
2 2D drawing	.29^**^									
3 Pattern assembly	.20^**^	.33^**^								
4 Elithorn mazes	.24^**^	.32^**^	.25^**^							
5 Mechanical reasoning	.20^*^	.50^**^	.28^**^	.42^**^						
6 Paper folding	.25^**^	.44^**^	.29^**^	.28^**^	.34^**^					
7 3D drawing	.28^**^	.50^**^	.23^**^	.29^**^	.34^**^	.42^**^				
8 Shape rotation	.08	.35^**^	.17^**^	.28^**^	.28^**^	.32^**^	.46^**^			
9 Perspective-taking	.18^**^	.44^**^	.19^**^	.21^**^	.39^**^	.25^**^	.35^**^	.26^**^		
10 Mazes	.20^**^	.39^**^	.23^**^	.33^**^	.42^**^	.38^**^	.41^**^	.38^**^	.29^**^	
11 KC total	.47^*^	.70^**^	.52^**^	.55^**^	.67^**^	.68^**^	.66^**^	.61^**^	.59^**^	.65^**^

*Note: * — p≤0.05. ** — p≤0.001; KC Total = total score for King’s Challenge battery*.

**Table S5:** Assumptions for factor analysis

Statistic	Whole sample	Science	Arts	Sports
KMO	.95	.93	.87	.91
Bartlett’s Chi-Square	5665.6	1434.68	534.81	534.81

*Note: Chi-Square p-value for all tracks was < .001*

**Table S6:** Split-Half reliability for all spatial tests for the whole sample and tracks

Test	N of items	Full sample		Science track		Arts track	
		S-HR	(SD)	S-HR	(SD)	S-HR	(SD)
Cross-sections	15	.87^**^	(.04)	.81**	(.05)	.84**	(.06)
2D drawing	5	.88**	(.07)	.81**	(.06)	.78**	(.09)
Pattern assembly	15	.80**	(.04)	.68**	(.04)	.76**	(.05)
Elithornmazes	10	.27*	(.01)	.23*	(.01)	.04*	(.02)
Mechanical reasoning	16	.67**	(.03)	.61**	(.04)	.47**	(.04)
Paper folding	15	.91**	(.04)	.84**	(.04)	.87**	(.07)
3D drawing	7	.95**	(.11)	.92**	(.11)	.92**	(.13)
Shape rotation	15	.88**	(.04)	.82**	(.05)	.81**	(.05)
Perspective - taking	15	.90**	(.06)	.90**	(.07)	.85**	(.07)
Mazes	10	.70**	(.03)	.62**	(.03)	.60**	(.05)

*Note: * = p≤0.05. ** = p≤0.001; S-HR = split-half reliability, SD = standard deviation for split-half reliability*.

**Table S7:** Bivariate correlations between SA and Raven’s within tracks

Test	Science N=482-503	Arts N=190-220	SportsN=222-259
Cross-sections	.33**	.26**	.22**
2D drawing	.36**	.38**	.52**
Pattern assembly	.37**	.32**	.30**
Elithorn mazes	.28**	.20**	.32**
Mechanical reasoning	.30**	.37**	.42**
Paper folding	.40**	.32**	.30**
3D drawing	.40**	.35**	.35**
Shape rotation	.38**	.31**	.33**
Perspective - taking	.17**	.28**	.30**
Mazes	.26**	.20**	.35**
KC total	.47**	.49**	.54**

*Note:* = p≤0.05. ** = p≤0.001; KC Total = total score for King’s Challenge battery*.

**Table S8:** Bivariate correlation between SA and Year grades for Mathematics and Russian language within tracks

Test	Science (N=509–547)			Arts (N=213–248)			Sports (N=212–267)		
	Math	Russian language	Fisher’s Z	Math	Russian language	Fisher’s Z	Math	Russian language	Fisher’s Z
Cross-sections	.15^**^	.05	ns	.18^**^	.04	ns	.10	–.07	ns
2D drawing	.10^*^	.03	ns	.26^**^	.12	ns	.24^**^	.09	ns
Pattern assembly	.18^**^	.03	ns	.15^*^	.14^*^	.29	.19^**^	.05	ns
Elithorn mazes	.15^**^	.04	ns	.03	.11	ns	.17^*^	.13^*^	ns
Mechanical reasoning	.12^**^	–.07	ns	.20^**^	.13^*^	.16	.20^**^	.08	ns
Paper folding	.15^**^	.05	ns	.21^**^	.16^*^	.48	.30^**^	.23^**^	1.24
3D drawing	.22^**^	.03	ns	.23^**^	.22^**^	.61	.20^**^	.17^**^	0.27
Shaperotation	.11^*^	–.02	ns	.07	.08	ns	.23^**^	.17^**^	1.01
Perspective taking -	.15^**^	.01	ns	.07	.01	ns	.11	.01	ns
Mazes	.14^**^	.02	ns	.12	.20^**^	–.22	.26^**^	.09	1.32
KC total	.22^**^	.02	ns	.25^**^	.19^**^	ns	.32^**^	.15^*^	ns

*Note:* = p≤0.05. ** = p≤0.001. NS = no Fisher’s z analysis was conducted when correlation(s) was non-significant*

**Table S9:** Bivariate correlations between SA and Exam for Mathematics and Russian language

Test	Whole sample N = 296 – 304		Science N = 190 – 200		Arts N = 76 – 92		Sports N = 10	
	Exam Math	Exam Rus	Exam Math	Exam Rus	Exam Math	Exam Rus	Exam Math	Exam Rus
Cross-sections	.24^**^	.11	.09	.16*	.08	–.06	.23	–.25
2D drawing	.20^**^	.11	–.04	.05	.13	.12	.52	–.01
Pattern assembly	.25^**^	.12^*^	–.01	.02	.2	.2	.21	.01
Elithorn mazes	.23^**^	.14^*^	.10	.05	.18	.28^**^	.16	–.17
Mechanical reasoning	.21^**^	.10	.02	.01	.07	.14	.21	.13
Paperfolding	.29^**^	.09	.02	–.01	.24^*^	.13	–.21	–.07
3D drawing	.29^**^	.13^*^	.14	.09	.18	.14	.15	–.48
Shape rotation	.27^**^	.10	.03	.01	.22	.19	–.12	–.11
Perspective - taking	.24^**^	.11	.11	.08	.2	.15	.01	–.52
Mazes	.24^**^	.15^**^	.03	.02	.25^*^	.34^**^	.15	.28
KC total	.34^**^	.16^**^	.08	.07	.30^**^	.27^*^	.14	–.15

*Note: * — p≤0.05. ** — p≤0.001. Fisher’s Z analysis showed no significant differences in SA correlations with Math vs. Russian Exam*.

**Table S10:** Outliers for three tracks for SA tests.

Test (number of items)	Science			Art			Sport		
	N	Number of outliers	Sample %	N	Number of outliers	Sample %	N	Number of outliers	Sample %
Cross-sections (15)	547	15	2.74	248	–	–	275	–	–
2D drawing (5)	529	18	3.40	243	8	3.29	270	–	–
Pattern assembly (15)	546	–	–	248	–	–	274	–	–
Elithorn mazes (10)	488	25	5.12	238	10	4.20	234	5	2.14
Mechanical reasoning (16)	546	8	1.47	246	–	–	274	–	–
Paper folding (15)	545	32	5.87	239	–	–	274	–	–
3D drawing (7)	521	–	–	229	–	–	270	21	7.78
Shape rotation (15)	532	4	0.75	226	–	–	269	–	–
Perspective – taking (15)	527	–	–	220	–	–	268	23	8.58
Mazes (10)	526	8	1.52	218	–	–	268	–	–
KC total (123)	526	3	0.57	218	1	0.46	267	2	0.75

*Note: KC Total = total scoress for King’s Challenge battery*.
